# How Selective Breeding Has Changed the Morphology of the American Mink (*Neovison vison*)—A Comparative Analysis of Farm and Feral Animals

**DOI:** 10.3390/ani11010106

**Published:** 2021-01-07

**Authors:** Anna Mucha, Magdalena Zatoń-Dobrowolska, Magdalena Moska, Heliodor Wierzbicki, Arkadiusz Dziech, Dariusz Bukaciński, Monika Bukacińska

**Affiliations:** 1 Department of Genetics, Wrocław University of Environmental and Life Sciences, 51-631 Wrocław, Poland; anna.mucha@upwr.edu.pl (A.M.); magdalena.moska@upwr.edu.pl (M.M.); heliodor.wierzbicki@upwr.edu.pl (H.W.); arkadiusz.dziech@upwr.edu.pl (A.D.); 2 Institute of Biological Sciences, Cardinal Stefan Wyszyński University in Warsaw, 01-938 Warsaw, Poland; d.bukacinski@uksw.edu.pl (D.B.); m.bukacinska@uksw.edu.pl (M.B.)

**Keywords:** American mink, comparative analysis, morphology, selective breeding

## Abstract

**Simple Summary:**

Decades of selective breeding carried out on fur farms have changed the morphology, behavior and other features of the American mink, thereby differentiating farm and feral animals. The uniqueness of this situation is not only that we can observe how selective breeding phenotypically and genetically changes successive generations, but also that it enables a comparison of farm minks with their feral counterparts. Such a comparison may thus provide valuable information regarding differences in natural selection and selective breeding. In our study, we found significant morphological differences between farm and feral minks as well as changes in body shape: trapezoidal in feral minks and rectangular in farm minks. Such a clear differentiation between the two populations over a period of several decades highlights the intensity of selective breeding in shaping the morphology of these animals and gives an indication of the speed of phenotypic changes and the species’ plasticity. This also suggests that the selective forces (selective breeding vs. natural selection) acting upon body dimensions of minks vary between feral and farm populations.

**Abstract:**

In this study, we performed a comparative analysis of the morphological traits between feral (*n* = 43) and farm (*n* = 200) individuals of the American mink in Poland to address the question of how multigenerational intensive selective breeding has morphologically differentiated these two populations. Nine body measurements and two proportion coefficients were obtained using adult individuals. The significance of differences between population means was assessed using the Wilcoxon test for independent samples, while the Kruskal–Wallis test was used to compare sex-population groups. Spearman’s correlation coefficients between measurements were estimated for each population. We also performed Principal Component Analysis (PCA) to identify the variables that were most closely correlated with variation in the trait measurements and to investigate the morphological differences between farm and feral minks. We found that the farm minks exhibited significantly higher mean values for eight out of eleven studied traits. Moreover, significant changes in forelimb length, with no concomitant changes in hindlimb length, were accompanied by differences in body shape: trapezoidal in feral minks and rectangular in farm minks. The PCA suggested an almost complete separation of the two populations and indicated that sexes were quite separate; farm males in particular constitute a wholly discrete cluster. Such a clear differentiation between the two populations and sexes over a period of several decades highlights the intensity of selective breeding in shaping the morphology of these animals.

## 1. Introduction

The American mink (*Neovison* *vison*) (hereafter: mink) is considered an invasive species in Europe. It originates from North America and its appearance in Europe is associated with the beginnings of the fur-farming industry in the 1920s [1]. Escapes of minks from fur farms led to the establishment of stable feral populations in many countries, mostly in northern Europe, e.g., Poland, Sweden, Norway and Belarus. Even though most fur farms are located in the northern parts of the continent, the mink is widely distributed, occurring in at least 28 European countries [2].

The mink has an impact on variety of prey species, e.g., water vole (*Arvicola amphibius*) or waterfowl, but also on other predators [3,4,5]. The mink is in competition with the European polecat (*Mustela putorius*) and the European mink (*Mustela* *lutreola*) because their niches overlap. Competition between American and European minks has resulted not only in changes in European mink distribution, but also in the body sizes of both species [6]; it is even considered to be one of the reasons for the European mink’s disappearance [5,7,8]. Nevertheless, despite its great plasticity and ability to dominate the European mink and polecat, American mink populations are declining in some regions of Europe [2]. 

The first fur farms in Poland were established in 1928, with their development intensifying after 1950. The first records of minks observed in the wild in this country date back to the 1950s [1]. While some feral individuals were probably escapees from farms, others must have been immigrants from neighbouring countries, like Belarus, where stable feral populations had already become established [9,10]. In Poland, the earliest breeding feral mink populations were probably established in the 1980s in the north-east of the country. Further expansion resulted in the colonization of the majority of Poland (except the south-east, where records of the mink are still scarce), with a greater abundance in the north [9]. 

Many years of selective breeding to improve the quality of fur-coat and pelt size, in line with market expectations [11,12], have changed the morphology, behaviour and other features of the species, thereby differentiating feral and farm animals [13,14]. Comparison of the genetic and morphological features between feral and domesticated individuals may thus provide valuable information regarding differences in natural selection and selective breeding, highlighting the principal features enabling feral minks to survive. This differentiation in morphological measurements has already been studied in different countries on the basis of skull and organ morphometrics, biochemical-genetics and genetic features [15,16,17,18,19,20,21]. Those analyses indicated that sexual dimorphism was less apparent in domesticated individuals, that feral animals were smaller than their farm equivalents, and that brain, heart and spleen were significantly smaller in domesticated individuals.

Genetic analysis of the mink populations in Denmark has shown a moderately high differentiation between feral and farm individuals [19]. In Poland, feral and farm minks constitute separate genetic clusters [22]. Moreover, genetic analyses and skull measurements indicate that there is often considerable differentiation within feral mink populations, which may be due to minimal gene flow between them, natural distribution differences or the divergent origins of individuals obtained for fur farming [18,23]. Nevertheless, in some regions of Europe their genetic differentiation is less noticeable [19]. 

Morphometric studies of feral mink have already been carried out [24]. However, to the best of our knowledge, no studies comparing body measurements between feral and farm individuals have been reported. Therefore, the main goal of our research was to perform a comparative analysis of the morphological traits between feral and farm individuals of the mink in Poland, and then to use the results to address the question of how multigenerational intensive selective breeding has morphologically differentiated these two populations. We hypothesize that the selective forces (selective breeding vs. natural selection) acting upon body weight and dimensions of minks vary between feral and farm populations, leading to phenotypic differences (increased body size of farm mink) and altered body proportions of individuals belonging to different groups.

## 2. Materials and Methods 

### 2.1. Data Collection

Body size measurements were obtained in 2010–2016. Only adult individuals from two mink populations were studied: feral (*n* = 43, 33 males and 10 females) and farm (*n* = 200, 100 males and 100 females). The animals from the feral population originated from two regions of Poland—the Lublin region (south-eastern Poland) and the Middle Vistula Valley (central-eastern Poland; the Project “Active protection of endangered island species in the Middle Vistula Valley area", financing agreement numbers: POIS.25.01.00-00-325/10 and 518/2013/USŁ/02/02). The feral minks were caught during the following periods: April to December 2013, April to December 2014, and January to December 2016. The farm minks (aged 9 months or older) came from one farm in the Wielkopolska region (western Poland). They were fed a standard diet of meat offal with the addition of vegetables and grains (according to their nutritional requirements) without any special supplements. There were no lactating females in both groups of mink (this could affect the mean body weight of the samples, see [25]). The mink farms operating in Poland follow the recommendations of the Polish Fur Breeders’ Association. The recommendations cover all breeding practices such as nutrition, veterinary care, selection criteria and housing conditions. Therefore, we believe that although the mink we studied came from one farm, they can be considered a representative group.

The following measurements were taken (using a tape measure to the nearest 1 mm or a scale with an accuracy of 1 g): body weight (BW; g), body length (BL; measured from the tip of the nose to the base of the tail, cm), breadth of chest (BC; measured behind the forelimbs, cm), tail length (TL; measured from the base of the tail to the last caudal vertebra, cm), height of the right ear (EH; measured from the base of the pinna to its tip, cm), length of the right fore limb (FRPL; measured from the axilla to the tips of the digits, cm), length of the right hind limb (RRPL; measured from the flank to the tips of the digits, cm), length of the right front paw (FRFL; measured from the tips of digits to the most strongly protruding bone in the proximal row of carpal bones, cm) and length of the right hind paw (RRFL; measured from the tips of the digits to the calcaneus, cm). We did not require a permit from the ethics committee to carry out the experiment on the animals, because all the measurements were taken post mortem. The measurements from the farm minks were collected after they had been killed at the end of the farm season (during pelting), while those from feral minks were collected after they had been caught and killed by hunters.

In addition, two proportion coefficients were estimated: PrCo1—the ratio of the length of the forelimb to the length of the hind limb—and PrCo2—the ratio of the length of the front paw to the length of the hind paw.

### 2.2. Statistical Analyses

Basic descriptive statistics, i.e., the median, arithmetic mean, standard deviation—SD—coefficient of variation—CV, were used to describe the measurements. The medians, means and their standard deviations (SD) were estimated to investigate the simultaneous effect of the population (feral or farm) and sex (male or female) on the trait measurements. The statistical significance of differences between populations and sexes was assessed using the Mann–Whitney test for independent samples. Moreover, four groups of animals were compared: farm females, feral females, farm males and feral males. The Kruskal–Wallis test, a non-parametric method for comparing two or more independent samples, was used in this analysis.

Spearman’s correlation coefficients between the trait measurements were estimated for each population and their significance was evaluated. The statistical significance of differences between correlation coefficients was assessed using the significance test of differences of two correlation coefficients. To do this, the confidence intervals for respective correlation coefficients were determined and then adjusted using Bonferroni method and the formula: 100%(1-α⁄k), where k is the number of correlations [26]. The correlation coefficients whose adjusted confidence intervals did not overlap were considered significantly different.

Principal component analysis (PCA) of the measurements and individuals was performed with *ade4* [27] and *factoextra* [28] packages to identify the variables that were most closely correlated with variation in the trait measurements and to investigate the morphological differences between farm and feral minks. The principal components with eigenvalues >1.0 were retained in the analysis [29]. This procedure for determining the number of PCA components that are above the noise level is called the Kaiser criterion [30].

All the statistical analyses were performed using the R package [31].

## 3. Results

To test the significance of the differences between the groups, we used non-parametric tests (e.g., the Mann–Whitney test) that examine the differences between the medians. However, if the groups have a similar distribution (this was the case in our study), it will shift the medians and means by the same amount, so the difference in the medians is the same as the difference in the means. Thus, the Mann–Whitney test is also a test of the difference in means [32]. Therefore, to indicate the significance of differences between the groups, we used means (with lowercase letters) to directly compare the results of our research with the results of other authors, which are mostly based on the comparison of means. Unequally sized groups (feral vs. farm) as well as the unbalanced female-to-male ratio in the feral mink diminish the statistical power. However, we used the non-parametric tests, which are less sensitive to unbalanced design. Furthermore, the smaller group (*n* = 43) is large enough to give reliable results.

The basic descriptive statistics of the mink populations are presented in Table 1. Farm individuals were characterized by overall greater values for BW, BL, BC, EH, FRPL and PrCo1. BW (1753 g in farm minks vs. 1127 g in feral minks, *p* < 0.05) and EH (1.65 cm in farm minks vs. 1.14 cm in feral minks, *p* < 0.05) were the most strongly differentiating traits between feral and farm individuals. On the other hand, the values of TL, RRPL, FRFL and RRFL were higher in the feral population. The differences for eight out of eleven traits (BW, BL, BC, TL, EH, FRPL, RRPL, PrCo1) were statistically significant. It is worth emphasizing that the farm minks had significantly longer fore limbs than their feral counterparts (11.54 vs. 9.43 cm, respectively), whereas there was not such a big difference in the hind limbs. Therefore, the body shapes of the two groups differed: that of the farm minks resembled a rectangle, whereas a trapezoidal body shape was characteristic of the feral individuals. These differences were also evident in the PrCo1 values.

Table 2 lists the basic descriptive statistics estimated for the sex groups in both populations. The highest values for eight out of eleven traits were found for farm males (BW, BL, BC, EH, FRPL, FRFL, RRFL and PrCo2). Feral males had the highest values of TL (17.72 cm) and RRPL (13.64 cm), while farm females had the highest values of PrCo1 (0.94). On the other hand, the lowest values for eight out of eleven traits were calculated for feral females (BW, BL, BC, EH, FRPL, RRPL, FRFL, RRFL); only TL (16.78 cm) was lower in farm females. PrCo2 (0.66) was equally low in both groups. The lowest value of PrCo1 (0.71) was estimated for feral males. In the case of BL (from 35.51 cm for feral females to 45.51 cm for farm males) and BC (from 15.04 cm for feral females to 21.48 cm for farm males), all groups differed significantly (*p* < 0.05). BW (from 795 g for feral females to 2287 g for feral males) significantly differentiated all the groups except farm females and feral males (1219 and 1227.27 g, respectively). Similarly, EH (from 1.09 to 1.89 cm) and FRPL (from 8.57 to 12.02 cm) were significantly different among all groups except feral females and males. TL, RRPL, FRFL and RRFL significantly differentiated the sexes, while PrCo1 (0.71–0.94) differed significantly between the same sexes, but between different sexes if they came from different populations. PrCo2 was significantly different (*p* < 0.05) only between farm males and farm females (0.7 and 0.66, respectively). 

Spearman’s correlation coefficients are set out in Table 3. The vast majority of the correlations between traits for farm individuals were statistically significant (44 out of 55). PrCo1 correlated significantly with FRPL and RRPL (0.51 and −0.28, respectively), while PrCo2 did so with BW, BC, FRFL, RRFL, RRFL and PrCo1 (0.21, 0.24, 0.22, 0.60, -0.23 and 0.24, respectively). The majority of significant correlations for farm minks took positive values (42 out of 44). Most of the negative correlations were not statistically significant and related to PrCo1. In comparison with farm individuals, there were only 19 significant correlations in the feral population and all took positive values. In both populations, BW and BL were most often positively and significantly correlated with other traits. Nevertheless, only two correlations significantly differentiated the populations: FRFL correlated significantly with EH and FRPL in farm individuals (0.58 and 0.62, respectively), whereas in the feral population these correlations were very weak and insignificant (0.01 and −0.08, respectively).

PCA of the data demonstrated three components (PC1, PC2, PC3) with eigenvalues >1.0, explaining 76.79% of the total variation in the data (Table 4). There was a big drop in eigenvalue between PC1 and PC2, and a smaller drop between PC2 and PC3. Together, PC1 and PC2 accounted for 64.07% of the total variance (46.46% and 17.61%, respectively). Thus, the first two components were retained for further analysis. All the factor loadings for PC1 were negative, but for PC2 five loadings were negative while the remainder were positive. The factor loadings for PC1 were rather high, exceeding 0.65 (absolute value). They ranged from −0.13 for PrCo1 to −0.92 for BW. The most strongly loaded on PC2 was PrCo1—proportion coefficient 1 (0.93). The other loadings ranged from 0.02 for BC to 0.55 for FRPL (positive values), and from −0.04 for BL to −0.52 for RRPL (negative values). 

**Figure 1:**
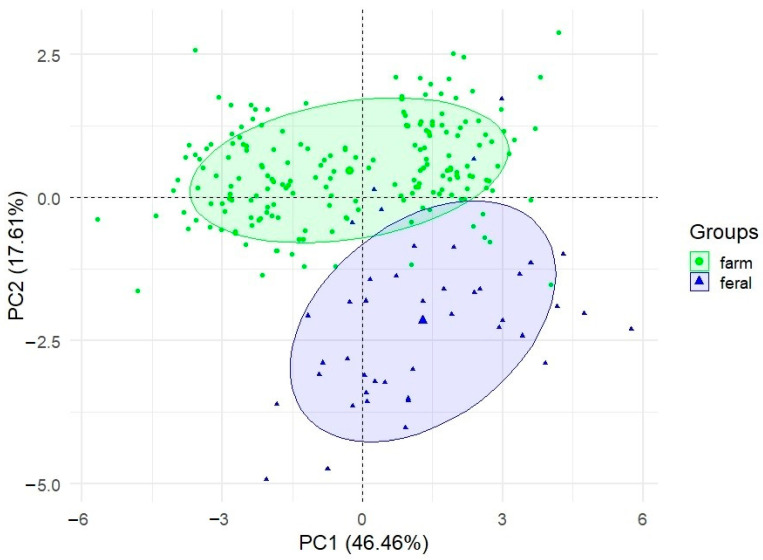
Principal component analysis (PCA) of 9 body measurements and 2 proportion coefficients (PrCo1 and PrCo2) exhibiting Table 1 and Table 2.

**Figure 2:**
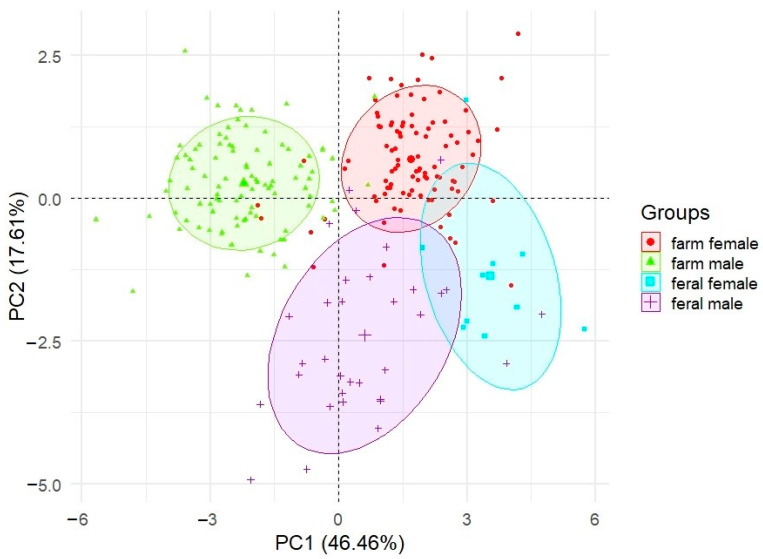
Principal component analysis (PCA) of 9 body measurements and 2 proportion coefficients (PrCo1 and PrCo2) of four sex-population groups: farm males, farm females, feral males and feral females. The axes represent loadings on components 1 and 2.

The PCA suggests almost complete separation of the two populations. The two-dimensional plot of the PC1 and PC2 axes in the traits and proportion coefficients (Figure 1) shows significant differences between the feral and farm mink populations and correlates with the results presented earlier in Table 1 and Table 2. Similar conclusions can be drawn by analysing the results of the PCA conducted for farm males, farm females, feral males and feral females (Figure 2). All the groups (sex × population) are quite separate; farm males in particular constitute a wholly discrete cluster.

## 4. Discussion

The establishment of fur farms and multi-generational selective breeding have provided exceptional conditions for studying the impact of evolutionary processes and domestication on a given species. The uniqueness of this situation is not only that we can observe how selective breeding phenotypically and genetically changes successive generations, but also that it enables comparison of farm minks with their feral counterparts. This study compared farm and feral American minks in order to investigate the early impact of domestication and selective breeding on mink morphology. Moreover, such a comparison highlights the importance of particular traits for the survival of the species when observations are made under conditions of reduced selection pressure (on farms) for traits related to activities necessary for survival, such as hunting and swimming.

Mean values of American mink body weight and measurements vary greatly. The average body weight and body length of the mink used in our study was comparable to the same measurements reported by Zalewski and Bartoszewicz [33], who conducted a study of feral mink in western Poland. They found that males’ body weight and length ranged from 1.00 to 1.70 kg and from 41 to 51 cm, respectively, while the same measurements for females ranged from 0.65 to 0.96 kg and from 36 to 44 cm, respectively. Comparing our results for feral individuals with those from other regions and countries leads to very different conclusions, however. Some studies have reported mean values of the traits similar to ours [6], whereas the results of other studies differ somewhat [24,34,35,36,37]. There is also a group of reports showing completely different results [38,39]. Overall, the greatest variety was documented for male BW (683.2 g–1521.1 g) and male BL (36.4 cm–65.31 cm). There were also considerable differences between farm minks. Mean BW for males and females in other studies conducted in Poland [40] were much higher than in our research (3020/3067 g for males, depending on the month, and 1773 g for females). However, it is important to note that all the above studies provided data relating mainly to BW and BL, sometimes TL or other traits. To the best of our knowledge, there are no reports apart from this paper describing the same range of traits that we measured and compared between farm and feral mink populations. 

According to Melero et al. [35], this variety of BW and BL in feral individuals is a result of three main factors: prey availability, sexual selection and energy waste. The impact of diet was reported by Zalewski and Bartosiewicz [33], who compared results from different countries and found that populations with lower BW and BL also had a much lower percentage of large prey in the diet. The importance of diet and prey availability for BW was described in another paper by the same authors [1]. In addition, the different origins of individuals in fur farming may be a factor where the genetic background has a significant influence on the size of the offspring, despite the ecological aspects. Such a relationship was suggested by Cavallini [41], who studied morphometric differences in the red fox (*Vulpes* *vulpes*) in central Italy. This author suggested that body size differences might be related to phylogenetic distances rather than to differences in ecological conditions. Furthermore, there appears to be no link between the size of feral minks and the climate they thrive in: individuals achieve high values of BW and BL in both warm and cool climates, and even in the same climate, minks can vary widely (see [33]). However, according to these latter authors, female BW decreases with increasing latitude (in contradiction of Bergmann’s rule). In addition, Zalewski and Brzeziński [1] stated that the BW of male escapees from fur farms was higher in the first few years of their appearance in the wild, only decreasing in later years. On the other hand, such differences regarding farm populations might be a result of different genetic backgrounds or different diets. Another important factor is how long the farm has been in operation. In older farms, individuals would be subjected to selective breeding for much longer periods, so the differences between their morphometrics compared with feral individuals would probably be greater than in more recently established farms. 

Much higher values of body dimensions in farm minks (BW, BL, BC, EH) are to be expected, as they have been selectively bred for those traits. Nevertheless, there are other factors that could influence the body dimensions of farm minks. As suggested by Lord et al. [42], who questioned the legitimacy of conclusions drawn from Belyaev’s experiment [43,44], many morphological features that are connected with domestication have insufficient scientific support for such a relationship. The morphological changes observed in domesticated species may not be mainly due to selective breeding, but may have other sources, e.g., an anthropogenic/farm diet or living in an anthropogenic environment. In our considerations, therefore, we include not only the effect of selective breeding on bigger body dimensions, but also the specific conditions associated with living on a farm.

Increased body size could also be a result of the lack of hunting opportunities and overall less activity, leading to less energy wasted. Moreover, the fodder provided by breeders is not as limited as in natural conditions and could differ from that available to feral minks. Niche overlap between the sexes is not a problem because of the abundance of fodder provided by the animal keepers. With regard to the size differences between the fore and hind limbs, the body shape of farm minks differs significantly from that of feral minks. Such changes are also evident in the different values of PrCo1, which is much higher for farm individuals. The more trapezoidal shape of feral minks could increase the effectiveness of hunting, while the lack of such activity in farm minks may lead to selection being less restrictive against long forelimbs. Furthermore, the body shape of minks is important for locomotion, both on land and in water, and has an impact on body drag during swimming [45]. Depriving farm minks of swimming opportunities might be another factor reducing the selection pressure, resulting in body shape differentiation. Finally, feed contamination or diet supplementation can induce changes in bone development in female minks and their offspring [46,47]. This can also be an important factor in differentiating the shape and body proportions of farm and feral mink.

Comparable changes in body size and shape were also documented in the red fox by Zatoń-Dobrowolska et al. [14]. These authors studied the morphometric differentiation between wild and farm foxes and reported similar results: eight out of 11 studied traits took significantly higher values in farm individuals. Sulik et al. [48] reported that measurements of imprints of metacarpal pads left by minks from farm and feral populations were significantly differentiated (much larger in farm minks), indicating great differences between the forelimbs size in both populations. This finding is consistent with the results of the present study. No differences in PrCo2 suggest that paw length could be less important for American minks for hunting or locomotion.

There are interesting differences in the correlations between EH and BW, BL, BC, FRFL, RRPL, RRFL. In farm individuals, these correlations are statistically significant and positive (ranging from 0.53 to 0.74), whereas in feral minks they are weak and non-significant (ranging from 0.01 to 0.33). The presence of such correlations in farm populations could be a natural consequence of selective breeding aimed at obtaining larger pelts: the larger the body size, the longer the ear—a so-called correlated response. On the other hand, it can be presumed that the absence of such correlations in feral populations could be due to natural selection favouring smaller ears: small ears could be beneficial when swimming, as little water can enter the earlobe. Therefore, even with a larger body size, small ears may be favoured by natural selection, resulting in the observed differences between correlations. Similarly, there is a strong and significant correlation in farm minks between FRPL and RRPL, whereas no such correlation exists in feral individuals. The probable explanation for this difference is that the trend towards forelimb shortening does not affect paw size, because this is not as crucial for hunting or locomotion as forelimb length, which governs the body shape. 

The results of the PCA listed in Table 1 and Table 2 emphasize the strong differences between the sexes as well as between feral and farm populations. The visible clustering of farm and wild mink, as well as the very clear sex separation (especially in farm mink) is probably due to two main reasons. First, body weight and pelt length are traits of relatively high heritability ranging from 0.43 to 0.49, and from 0.42 to 0.45, respectively [49,50]. Intensive selective breeding aimed at improving these traits (especially in the group of farm males) has led to a significant differentiation between farm and wild mink. Second, American minks are naturally sexually dimorphic, with males being up to twice the size of females [34]. It therefore seems, that naturally occurring sexual dimorphism, together with intensive selective breeding, led to clustering, both between populations and between sexes. 

Our results contrast with those obtained by Hammershøj et al. [51], who divided feral minks from Denmark into farm and wild individuals. In their research, about 80% of the Danish minks captured in the wild were escapees from farms. If the situation in Poland was similar, the farm and feral populations from our study would not have been so widely differentiated. Moreover, according to Zalewski et al. [22], who studied feral and farm minks in Poland using 14 microsatellite markers, only 17% of feral minks were assigned to the farm clusters. A recent study conducted in Denmark [37], differentiating wild and farm individuals using body length differences and tetracycline as a biomarker, yielded a much lower percentage of farm individuals in feral populations than in the study by Hammershøj et al. [51]. Therefore, such results underline the importance of carrying out appropriate studies prior to concluding whether feral minks actually thrive in the wild, or whether they are mostly escapees. This would enable the necessary steps to be taken to manage feral populations. If the majority of feral minks are farm escapees, management should focus on limiting escapes from fur farms using relevant biosecurity measures. On the other hand, if stable feral populations already exist, wildlife and fur breeder organizations should focus more on limiting their numbers in the wild. 

In recent months, coronavirus outbreaks at mink farms in the US and in Europe have killed thousands of individuals. Outbreaks have been detected in Denmark, Sweden, Spain, the Netherlands, Italy and the United States [52]. The first cases of the coronavirus in farm minks have also been reported in Poland [53]. This raises concerns that the infection could spread between farm and feral minks (farm escapees could pass the virus to feral populations). In this context, the results of our research (significant differences in morphometrics between farm and feral minks) could be used as one of the tools for monitoring farm escapes. Farm escapees (potential coronavirus vectors), having significantly larger body sizes, could be caught, which would reduce the risk of infecting feral minks.

## 5. Conclusions

Selective breeding can be considered an important factor influencing the morphology of American minks. Our research proved that the farm minks had significantly greater measurements for nine out of the eleven studied traits. Moreover, significant changes in forelimb length, with no concomitant changes in hindlimb length, were accompanied by differences in body shape: trapezoidal in feral minks and rectangular in farm minks. As the studied traits are important for hunting and locomotion, the non-necessity or lack of opportunities for such activities in farm individuals could significantly alter selection pressure for these traits, resulting in the recorded differences. Such a clear differentiation between the two populations over a period of several decades highlights the intensity of selective breeding in shaping the morphology of these animals and gives an indication of the speed of phenotypic changes and the species’ plasticity. Despite the fact that only a few decades have passed between the first escapes from fur farms and the establishment of a feral population in Poland, such significant morphological differences are already measurable between the two populations. Research on the genetic structure of both mink populations should shed further light on the genetic background of these morphological differences.

## Figures and Tables

**Table 1:** Basic descriptive statistics of the studied traits of feral and farm minks.

	Trait										
	BW (g)	BL (cm)	BC (cm)	TL (cm)	EH (cm)	FRPL (cm)	RRPL (cm)	FRFL (cm)	RRFL (cm)	PrCo1	PrCo2
FARM MINK											
*n* median	200 1725.00	200 42.00	200 19.00	195 18.00	199 1.70	200 11.00	199 13.00	200 4.00	199 6.00	199 0.92	199 0.67
mean	1753.00 ^a^	42.38 ^a^	19.18 ^a^	18.19 ^a^	1.65 ^a^	11.54 ^a^	12.51 ^a^	4.26	6.28	0.93 ^a^	0.68
SD	607.71	3.82	2.77	3.55	0.30	1.09	1.15	0.57	0.68	0.08	0.08
VC (%)	34.67	9.02	14.43	19.53	18.16	9.45	9.19	13.48	10.90	8.33	12.12
FERAL MINK											
*n* median	43 1200.00	43 41.00	43 17.00	43 19.70	43 1.00	43 9.40	43 13.30	43 4.50	43 6.40	43 0.70	43 0.69
mean	1126.74 ^b^	40.27 ^b^	17.10 ^b^	19.44 ^b^	1.14 ^b^	9.43 ^b^	13.15 ^b^	4.31	6.39	0.72 ^b^	0.67
SD	275.02	3.76	2.36	1.97	0.34	1.59	1.54	0.82	0.71	0.11	0.09
VC (%)	24.41	9.33	13.78	10.16	29.37	16.82	11.72	18.92	11.11	14.61	13.32

BW—body weight; BL—body length; BC—breadth of chest; TL—tail length; EH—height of the right ear; FRPL—length of the right fore limb; RRPL—length of the right hind limb; FRFL—length of the right front paw; RRFL—length of the right hind paw; PrCo1—proportion coefficient 1; PrCo2—proportion coefficient 2. *n* = number of individuals, SD = standard deviation, VC = variation coefficient. a, b—means differing significantly (*p* < 0.05) in the columns are marked with different lowercase letters.

**Table 2:** Basic descriptive statistics of the studied traits in farm females, feral females, farm males and feral males.

	Trait										
	BW (g)	BL (cm)	BC (cm)	TL (cm)	EH (cm)	FRPL (cm)	RRPL (cm)	FRFL (cm)	RRFL (cm)	PrCo1	PrCo2
FARM FEMALES											
*n* median	100 1200.00	100 39.00	100 17.00	96 17.00	100 1.45	100 11.00	99 12.00	100 4.00	99 6.00	99 0.92	99 0.67
mean	1219.00 ^b^	39.24 ^a^	16.88 ^a^	16.78 ^a^	1.42 ^b^	11.06 ^b^	11.81 ^a^	3.87 ^a^	5.87 ^a^	0.94 ^a^	0.66 ^b^
SD	219.59	2.24	1.47	3.00	0.19	0.94	0.97	0.42	0.59	0.08	0.09
VC (%)	18.01	5.71	8.72	17.85	13.39	8.53	8.18	10.80	10.08	8.21	13.44
FERAL FEMALES											
*n* median	10 750.00	10 35.65	10 15.00	10 17.25	10 1.00	10 8.50	10 11.35	10 4.15	10 5.85	10 0.72	10 0.69
mean	795.00 ^c^	35.51 ^b^	15.04 ^b^	17.31 ^a^	1.09 ^c^	8.57 ^c^	11.54 ^a^	3.82 ^a^	5.79 ^a^	0.75 ^b^	0.66 ^ab^
SD	138.34	2.38	1.91	1.97	0.51	1.02	0.94	0.67	0.43	0.10	0.09
VC (%)	17.40	6.69	12.73	11.39	46.67	11.87	8.12	17.49	7.39	12.97	13.66
FARM MALES											
*n* median	100 2250.00	100 46.00	100 21.00	99 20.00	99 1.90	100 12.00	100 13.00	100 4.50	100 7.00	100 0.92	100 0.71
mean	2287.00 ^a^	45.51 ^c^	21.48 ^c^	19.57 ^b^	1.89 ^a^	12.02 ^a^	13.20 ^b^	4.64 ^b^	6.69 ^b^	0.91 ^a^	0.70 ^a^
SD	343.60	2.12	1.59	3.53	0.18	1.01	0.86	0.43	0.50	0.08	0.07
VC (%)	15.02	4.67	7.42	18.02	9.67	8.44	6.55	9.33	7.49	8.25	10.36
FERAL MALES											
*n* median	33 1250.00	33 42.00	33 17.00	33 20.00	33 1.10	33 9.50	33 14.00	33 4.60	33 6.70	33 0.68	33 0.69
mean	1227.27 ^b^	41.71 ^d^	17.72 ^d^	20.08 ^b^	1.16 ^c^	9.70 ^c^	13.64 ^b^	4.46 ^b^	6.58 ^b^	0.71 ^b^	0.68 ^ab^
SD	221.53	2.78	2.13	1.48	0.27	1.65	1.35	0.81	0.68	0.11	0.09
VC (%)	18.05	6.66	12.00	7.35	23.46	16.98	9.88	18.07	10.34	15.14	13.36

BW—body weight; BL—body length; BC—breadth of chest; TL—tail length; EH—height of the right ear; FRPL—length of the right fore limb; RRPL—length of the right hind limb; FRFL—length of the right front paw; RRFL—length of the right hind paw; PrCo1—proportion coefficient 1; PrCo2—proportion coefficient 2 *n* = number of individuals, SD = standard deviation, VC = variation coefficient a,b,c,d—means differing significantly (*p* < 0.05) in the columns are marked with different lowercase letters.

**Table 3:** Spearman’s correlation coefficients between the studied traits of feral (lower triangle; 43 individuals) and farm (upper triangle; 200 individuals) minks.

Trait	BW	BL	BC	TL	EH	FRPL	RRPL	FRFL	RRFL	PrCo1	PrCo2
BW	1.00	0.87 *	0.94 *	0.57 *	0.74 *	0.55 *	0.69 *	0.69 *	0.63 *	−0.05	0.21 *
BL	0.87 *	1.00	0.80 *	0.56 *	0.72 *	0.57 *	0.70 *	0.71 *	0.67 *	−0.05	0.19
BC	0.78 *	0.59 *	1.00	0.51 *	0.72 *	0.46 *	0.64 *	0.67 *	0.57 *	−0.10	0.24 *
TL	0.52 *	0.62 *	0.32	1.00	0.48 *	0.39 *	0.52 *	0.47 *	0.48 *	−0.08	0.11
EH	0.31	0.33	0.21	0.17	1.00	0.35 *	0.54 *	0.58 *^a^	0.53 *	−0.11	0.17
FRPL	0.35	0.40	-0.08	0.33	0.20	1.00	0.63 *	0.62 *^a^	0.55 *	0.51 *	0.22 *
RRPL	0.60 *	0.61 *	0.31	0.56 *	0.17	0.53 *	1.00	0.57 *	0.65 *	−0.28 *	0.05
FRFL	0.44	0.46	0.64 *	0.54 *	0.01 ^b^	−0.08 ^b^	0.29	1.00	0.60 *	0.17	0.60 *
RRFL	0.65 *	0.62 *	0.70 *	0.64 *	0.04	0.18	0.60 *	0.79 *	1.00	−0.04	−0.23 *
PrCo1	−0.08	−0.07	−0.35	−0.10	0.14	0.71 *	−0.17	−0.34	−0.32	1.00	0.24 *
PrCo2	−0.01	0.03	0.24	0.24	−0.04	−0.38	−0.19	0.69 *	0.17	−0.24	1.00

BW—body weight; BL—body length; BC—breadth of chest; TL—tail length; EH—height of the right ear; FRPL—length of the right fore limb; RRPL—length of the right hind limb; FRFL—length of the right front paw; RRFL—length of the right hind paw; PrCo1—proportion coefficient 1; PrCo2—proportion coefficient 2; Correlation coefficients marked with * are significant at *p* < 0.05; Corresponding correlation coefficients with statistically significant differences (*p* < 0.05) are marked with different lowercase letters.

**Table 4:** The first three principal components (PC1, PC2 and PC3), which account for more than 76% of the total variation from the principal component analysis. The loadings indicate how much each trait contributes to a particular PC and whether they are positively or negatively correlated.

Traits	PC1	PC2	PC3
BW	−0.92	0.14	−0.05
BL	−0.91	−0.04	−0.07
BC.	−0.87	0.02	0.07
TL	−0.47	−0.33	0.00
EH	−0.68	0.41	−0.16
FRPL	−0.65	0.55	−0.20
RRPL	−0.67	−0.52	−0.18
FRFL	−0.73	−0.19	0.54
RRFL	−0.71	−0.38	−0.30
PrCo1	−0.13	0.93	−0.07
PrCo2	−0.23	0.14	0.95
Eigenvalue	5.11	1.94	1.40
Percentage variance	46.46	17.61	12.72
Percentage cumulative variance	46.46	64.07	76.79

BW—body weight; BL—body length; BC—breadth of chest; TL—tail length; EH—height of the right ear; FRPL—length of the right fore limb; RRPL—length of the right hind limb; FRFL—length of the right front paw; RRFL—length of the right hind paw; PrCo1—proportion coefficient 1; PrCo2—proportion coefficient 2.

## Data Availability

The data presented in this study are available on request from the corresponding author.
